# Applications of Artificial Intelligence in Pediatric Anesthesia: A Structured Narrative Review

**DOI:** 10.7759/cureus.102535

**Published:** 2026-01-29

**Authors:** Aditya Shah, Patrick Fakhoury, Emma Butler, Misha Patel, Caleb Zimmerman, Lewis Macdonald, Aiman Almasnaah, Deepti Sanku, Kush Patel, Wael Saasouh

**Affiliations:** 1 College of Medicine, Central Michigan University, Mount Pleasant, USA; 2 Department of Biomedical Sciences, Grand Valley State University, Allendale, USA; 3 School of Medicine, Wayne State University, Detroit, USA

**Keywords:** artificial intelligence, decision support systems, machine learning, patient safety, pediatric anesthesia

## Abstract

Artificial intelligence (AI) and machine learning (ML) are emerging as valuable tools in pediatric anesthesia practice. This narrative review examines applications across airway management, intraoperative monitoring, and postoperative care. Following a systematic literature search of four databases through 2024, 11 studies were analyzed examining AI methodologies in pediatric anesthesia settings. The review found that ML models consistently demonstrated improved predictive performance within studied datasets compared with traditional clinical approaches, particularly in endotracheal tube sizing and placement, hypoxemia prediction, and pain assessment. Models achieved high predictive accuracy rates and discrimination metrics across diverse clinical applications, with some demonstrating placement error reductions of 40-50% in retrospective analyses and achieving prediction accuracy exceeding 90%. Despite promising results, most studies were retrospective single-center analyses, highlighting the need for prospective multi-center validation and implementation research. Key challenges include ensuring model generalizability across diverse populations, integrating AI into clinical workflows, addressing regulatory requirements, and maintaining transparency in decision-making processes. Future work should focus on external validation, improving model interpretability, and developing frameworks for responsible integration into pediatric anesthesia practice to maximize patient safety benefits while addressing ethical considerations.

## Introduction and background

The integration of artificial intelligence (AI) and machine learning (ML) into pediatric anesthesia represents a paradigm shift in clinical practice, offering opportunities to enhance patient safety, optimize procedural efficiency, and refine clinical decision-making. Pediatric general anesthesia presents unique challenges distinct from adult practice, including significant anatomical variability across age groups, smaller margins for error in airway management, rapid physiological changes during development, and the frequent inability of young patients to communicate symptoms or cooperate with procedures. These factors make standardized approaches particularly challenging and highlight the potential value of individualized, data-driven decision support.

Traditional anesthetic management relies predominantly on predefined formulas and population-based protocols that are often unifactorial and assessed at single time points [1]. In contrast to these conventional tools, AI and ML approaches offer the potential to incorporate multiple patient-specific variables simultaneously and may identify patterns that are not readily apparent through standard clinical assessment. Emerging evidence demonstrates the capacity of AI models to improve accuracy in critical clinical applications, including endotracheal tube (ETT) size and depth prediction, intraoperative hypoxemia risk stratification, and postoperative pain assessment, thereby facilitating improvements in airway management, intraoperative monitoring, and postoperative care [2,3].

Postoperative pain assessment holds particular significance for pediatric populations, where traditional evaluation methods face inherent limitations. Self-reporting, while considered the gold standard, proves inadequate for many pediatric patients. This is especially true for those with intellectual disabilities, developmental delays, or age-related communication barriers who cannot reliably articulate their pain experience. AI-driven pain assessment tools offer increased objectivity and consistency in evaluating these vulnerable populations, addressing a critical gap in pediatric perioperative care [2-6].

Despite its clinical promise, the implementation of AI in pediatric anesthesia faces substantial challenges. Data standardization across institutions, external validation of predictive models, and clinician adoption remain significant barriers to widespread integration. Furthermore, regulatory frameworks and ethical considerations surrounding AI-driven decision-making in pediatric care require careful deliberation. Addressing these challenges will necessitate multi-center validation trials, prospective implementation studies, and the development of integration frameworks that align AI technologies with established clinical workflows. This narrative review synthesizes current evidence on AI applications in pediatric anesthesia, examining methodological approaches, clinical performance metrics, and implementation barriers. By evaluating the state of the field and identifying critical knowledge gaps, this review aims to inform future research directions and guide the responsible integration of AI technologies into pediatric anesthetic practice.

## Review

Methods

Search Strategy

A comprehensive systematic literature search was conducted across four major electronic databases: EBSCO (Elton B. Stephens Company), PubMed, Scopus, and Web of Science. The search included studies published up to 2024, as the literature search was completed in late 2024. The search strategy utilized Medical Subject Headings (MeSH) terms and keyword combinations tailored to identify studies examining artificial intelligence and machine learning applications in pediatric anesthesia.

Database-specific search strategies were developed to capture relevant literature across all platforms. Each database employed a combination of controlled vocabulary terms and free-text keywords related to anesthesia, AI and ML, and pediatric populations. The initial search yielded 643 records: 17 from EBSCO, 122 from PubMed, 108 from Scopus, and 396 from Web of Science. The complete search syntax for each database is presented in Table 1.

**Table 1:** Database-Specific Search Strategies

Database	Search Strategy
EBSCO	((MH Anesthesia+) OR (MH Anesthesiologists+) OR (TI anesthesi* OR AB anesthesi*) OR (MH "Pediatric Anesthesia+")) AND ((MH "Artificial Intelligence+") OR (MH "Machine Learning+") OR (TI "artificial intelligence" OR AB "artificial intelligence") OR (TI "machine learning" OR AB "machine learning")) AND ((MH Pediatrics+) OR (MH Adolescent+) OR (MH Child+) OR (MH Infant+) OR (TI pediatric* OR AB pediatric*) OR (TI adolescen* OR AB adolescen*) OR (TI child* OR AB child*) OR (TI infant* OR AB infant*))
PubMed	("Anesthesia"[Mesh] OR "Anesthesiologists"[Mesh] OR anesthesi*[tiab] OR "Pediatric Anesthesia"[Mesh]) AND ("Artificial Intelligence"[Mesh] OR "Machine Learning"[Mesh] OR "artificial intelligence"[tiab] OR "machine learning"[tiab]) AND ("Pediatrics"[Mesh] OR "Adolescent"[Mesh] OR "Child"[Mesh] OR "Infant"[Mesh] OR pediatric*[tiab] OR adolescen*[tiab] OR child*[tiab] OR infant*[tiab])
Scopus	(INDEXTERMS(Anesthesia) OR INDEXTERMS(Anesthesiologists) OR TITLE-ABS(anesthesi*) OR INDEXTERMS("Pediatric Anesthesia")) AND (INDEXTERMS("Artificial Intelligence") OR INDEXTERMS("Machine Learning") OR TITLE-ABS("artificial intelligence") OR TITLE-ABS("machine learning")) AND (INDEXTERMS(Pediatrics) OR INDEXTERMS(Adolescent) OR INDEXTERMS(Child) OR INDEXTERMS(Infant) OR TITLE-ABS(pediatric*) OR TITLE-ABS(adolescen*) OR TITLE-ABS(child*) OR TITLE-ABS(infant*))
Web of Science	(Anesthesia OR Anesthesiologists OR anesthesi* OR "Pediatric Anesthesia") AND ("Artificial Intelligence" OR "Machine Learning") AND (Pediatrics OR Adolescent OR Child OR Infant OR pediatric* OR adolescen* OR child* OR infant*)

The PICO (Population, Intervention, Comparison, Outcome) framework guided the development of the search strategy and study selection criteria. The population included pediatric patients undergoing anesthesia procedures, the intervention comprised artificial intelligence and machine learning applications, the comparison involved traditional clinical methods and formulas, and outcomes focused on prediction accuracy, clinical performance metrics, and patient safety indicators. The complete PICO framework is detailed in Table 2.

**Table 2:** PICO Framework

Component	Description
Population (P)	Pediatric patients including infants, children, and adolescents (age 0-18 years) undergoing anesthesia procedures
Intervention (I)	Artificial intelligence and machine learning applications in pediatric anesthesia practice, including predictive models, decision support systems, and automated monitoring tools
Comparison (C)	Traditional clinical methods, including age-based formulas, height-based formulas, standard monitoring systems, and conventional clinical assessment tools
Outcome (O)	Primary outcomes: accuracy of endotracheal tube size and depth prediction, hypoxemia prediction performance, postoperative pain assessment accuracy, airway device classification accuracy, and perioperative risk stratification Secondary outcomes: clinical decision-making efficiency, patient safety metrics, prediction sensitivity and specificity, and model validation performance

Eligibility Criteria

Studies were selected based on predetermined inclusion and exclusion criteria to ensure relevance and quality for this narrative review.

Inclusion criteria comprised: (i) studies investigating AI or ML applications in pediatric anesthesia practice, (ii) research involving human pediatric participants, including children, adolescents, and infants, (iii) peer-reviewed publications, and (iv) full-text articles available in English.

Exclusion criteria included: (i) non-peer-reviewed sources such as conference abstracts, editorials, and opinion pieces, (ii) publications in languages other than English, (iii) studies not addressing the intersection of anesthesia, AI, and pediatric populations, and (iv) animal or in vitro studies without clinical human application.

Study Selection Process

Two independent reviewers conducted the study selection process using Rayyan, a web-based systematic review management tool [7]. Initial screening involved removing duplicates using Rayyan's automated deduplication feature, which identified and removed 108 exact duplicates, leaving 535 unique records for screening.

Title and abstract screening was performed independently by both reviewers to identify potentially relevant studies. Subsequently, full-text articles of potentially eligible studies were retrieved and assessed against the predetermined eligibility criteria. Disagreements between reviewers were resolved through discussion, with arbitration by a third independent reviewer when consensus could not be reached.

**Figure 1:**
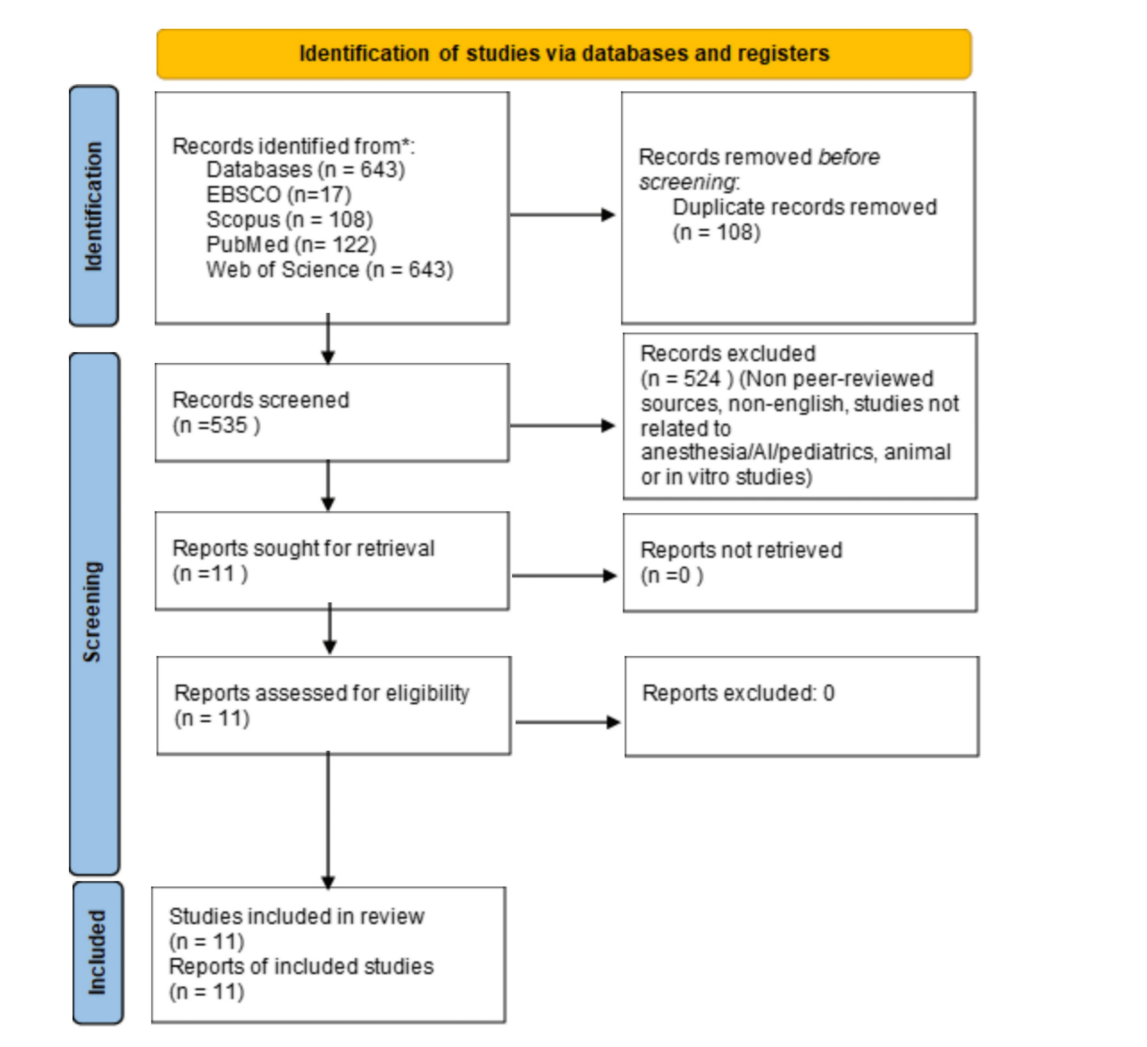
PRISMA Diagram PRISMA = Preferred Reporting Items for Systematic Reviews and Meta-Analyses

This process resulted in 524 exclusions based on the following criteria: non-peer-reviewed sources, language limitations, studies unrelated to the core topic of anesthesia/AI/pediatrics, and animal or in vitro studies. Ultimately, 11 studies met all inclusion criteria and were included in the narrative review. The study selection process is illustrated in Figure 1.

Data Extraction and Management

A standardized data extraction form was developed to ensure consistent collection of relevant information from included studies. Two reviewers independently extracted data, with discrepancies resolved through consensus discussion. The following variables were systematically extracted from each study: Study Design, Target Population, AI/ML Methods, Key Findings, Validation, and Statistical Metrics. The extracted data were compiled into a comprehensive summary table, presenting the characteristics and key findings of all included studies [8-18].

Data Synthesis and Analysis

Although systematic search and screening procedures were applied, this work is intended as a structured narrative review. The use of PRISMA-style reporting elements, dual reviewer screening, and standardized data extraction was adopted to improve methodological rigor and transparency of the narrative synthesis process. Given the heterogeneity of AI methodologies, outcome measures, and clinical applications across included studies, a narrative synthesis approach was employed for this review. As this is a narrative review rather than a systematic meta-analysis, the focus was on qualitative synthesis and thematic organization of findings. The performance of AI models was evaluated using reported statistical metrics, including accuracy, area under the receiver operating characteristic curve (AUROC), sensitivity, specificity, and mean absolute error (MAE).

The narrative synthesis examined the relative effectiveness of AI-based approaches compared to traditional clinical methods or standard predictive models, identified consistent patterns in performance outcomes across studies, assessed the clinical applicability and practical implications of findings, and highlighted gaps and limitations in the current evidence base. Findings were organized thematically according to clinical application areas to facilitate meaningful interpretation and identify future research directions in this evolving field.

Results

Study Characteristics

A total of 11 studies were identified, covering a spectrum of AI-based and ML-based applications in pediatric anesthesia. Study designs were primarily retrospective cohort or observational studies, with one prospective multicenter cohort study. Across these studies, investigators used diverse AI/ML methods, gradient boosting, support vector machines (SVMs), elastic net regression, Random Forest, and neural networks. Common outcomes of interest included endotracheal tube size and depth estimation accuracy, hypoxemia prediction, postoperative pain assessment, airway device classification, and automated ventilation control. Table 3 summarizes the characteristics of all included studies.

**Table 3:** Summary of included studies evaluating AI/ML applications in pediatric care. AI/ML = artificial intelligence/machine learning; ANN = artificial neural network; ASA = American Society of Anesthesiologists; AUROC = area under the receiver operating characteristic curve; AUPRC = area under the precision-recall curve; BiPAP = bi-level positive airway pressure; BPPI = Bochum Postoperative Pain Instrument; CHIPPS = Children's and Infants Postoperative Pain Scale; CI = confidence interval; DTR = decision tree regression; EHR = electronic health record; EMR = electronic medical record; EtCO2 = end-tidal carbon dioxide; ETT = endotracheal tube; GBR = gradient boosting regression; GBRT = gradient boosted regression trees; GBM = gradient boosting machine; LASSO = least absolute shrinkage and selection operator; LMA = laryngeal mask airway; LR = linear regression; LSTM = long short-term memory; MAE = mean absolute error; MSE = mean squared error; NN = neural network; NNA = number needed to alert; NPV = negative predictive value; PACU = post-anesthesia care unit; PPV = positive predictive value; PPPM-D = Parents Postoperative Pain Measure for Toddlers and Preschoolers; RF = Random Forest; RMSE = root mean square error; RNN = recurrent neural network; SVM = support vector machine; SVR = support vector regression; VAF = variance accounted for.

Citation	Study	Study Design	Target Population	AI/ML Methods	Key Findings	Validation	Statistical Metrics
Shim et al, 2021 [8]	Machine learning model for predicting the optimal depth of tracheal tube insertion in pediatric patients: A retrospective cohort	Retrospective cohort study evaluating ML algorithms for ETT depth prediction.	Pediatric surgical patients <7 years (Korean cohort).	Random Forest, Elastic Net, SVM, ANN trained on age, sex, height, weight.	Random Forest: 79.0% optimal placement vs. 44.4-66.9% for traditional formulas.	5-fold cross-validation; 30% test set.	Random Forest: 79.0% (95% CI: 73.5-83.6); Chi-squared tests (p < 0.001).
							Age-specific optimal placement rates (months): 38.3%, 50.6%, 52.3%, 58.5%, 60.9%, 64.4%, 72.5%, 76.6%, 77.0%, 77.4%.
Shim et al, 2023 [9]	Predicting the risk of inappropriate depth of endotracheal intubation in pediatric patients using machine learning	Retrospective cohort study assessing ML for ETT placement prediction	1,436 pediatric patients <7 years (Korean cohort).	Elastic Net regression trained on age, sex, height, weight.	83% correct placement vs. 35.7-62.2% for traditional methods.	Internal validation with retrospective datasets.	R² = 66.355; MAE = 0.743 cm; MSE = 1.000; VAF = 66.338. Test-set size: n=429; subgroup misplacement risk ranged 17.9-46.6% across age groups.
Im et al, 2022 [10]	Development of a deep learning model that predicts Bi-level positive airway pressure failure	Retrospective cohort study predicting BiPAP failure.	630 BiPAP sessions (27.7% failure rate).	LSTM-RNN trained on broad EMR feature set (301 variables)	LSTM RNN outperformed comparator models, with AUROC 0.81 at 6 hours and 0.84 at 24 hours in the overall cohort and AUROC 0.88 at 6 hours and 0.86 at 24 hours in the hypoxemic subgroup, and when operating at NNA 2 it identified nearly 80% of BiPAP failures within 6 hours.	Training and holdout test split of 80% and 20% with all sessions from a given patient kept in the same split.	AUROC 0.81 at 6 hours with 95% confidence interval 0.80 to 0.82, and 0.84 at 24 hours with 95% confidence interval 0.83 to 0.85
							Hypoxemic subgroup AUROC 0.88 at 6 hours and 0.86 at 24 hours; at 6 hours using number needed to alert of 2 the model identified nearly 80% of BiPAP failures
							Number needed to alert is the inverse of positive predictive value; median time on BiPAP prior to intubation was 32.8 hours in the failure group
Kim et al, 2023 [11]	Predicting optimal endotracheal tube size and depth in pediatric patients using demographic data and ML	Retrospective cohort study using Gradient Boosted Regression Trees.	37,057 children <12 years.	GBRT trained on demographics (age, height, weight).	ETT size exact-match 58.2%/70.1% (uncuffed/cuffed) and ±0.5 mm accuracy 98.1%/99.5%; depth MAE 0.71–0.72 cm vs formula-based methods.	Internal (retrospective) validation using a temporal split	Macro-averaged F1 (ETT size): 0.502 (uncuffed), 0.669 (cuffed); MAE (depth): 0.71–0.72 cm; plus, exact-match and ±0.5 mm accuracy
Park et al, 2023 [12]	Machine learning-based prediction of intraoperative hypoxemia for pediatric patients	Retrospective observational study using prospectively collected intraoperative vital signs and anesthesia ventilator machine parameters	13,130 pediatric cases (<18 years)	Gradient boosting machine, long short-term memory, and transformer models trained on ventilator and monitor data extracted every 2 seconds using 1-minute segments to predict whether oxygen saturation will drop below 95% in the upcoming 1 minute	In the model development test dataset, gradient boosting achieved AUROC 0.904, higher than long short-term memory AUROC 0.843 and transformer AUROC 0.897. In the temporal holdout validation cohort, gradient boosting achieved AUROC 0.939 with sensitivity 0.855 and specificity 0.807, which was higher than long short-term memory AUROC 0.904 and transformer AUROC 0.929	Internal test dataset for model comparison plus a temporal holdout validation cohort	Outcome definition was oxygen saturation below 95% at any point during surgery. Model development set included 1,540 patients with hypoxemia out of 13,130 and 2,367 hypoxemia episodes.
							Holdout validation used 1,510 patient records with 200 hypoxemic patients and 289 episodes. AUROC and AUPRC in holdout were 0.939 and 0.235 for gradient boosting, 0.904 and 0.124 for long short-term memory, 0.929 and 0.145 for transformer
Gude et al, 2024 [13]	New postoperative pain instrument for toddlers: Secondary analysis after tonsil surgery	Retrospective observational study using Boruta and symbolic regression.	149 toddlers post-tonsillectomy (1,067 pain assessments).	Boruta selected variables; symbolic regression simplified scoring.	Rescue medication indications occurred in 19.96% of assessments and symbolic regression produced a four predictor BPPI rule with 94.94% cross validated accuracy using CHIPPS at least 4 or PPPM D item less energy than usual plus either cries more easily than usual or groans or moans more than usual	Internal validation using a 75% train and 25% test cross validation approach, with Boruta bootstrapping used to prioritize stability	Cross validated accuracy 94.94% and Boruta importance scores including 0.94 for PPPM D score and 0.94 for CHIPPS score
Zhou et al, 2022 [14]	Prediction of endotracheal tube size in pediatric patients: Development and validation of ML models	Retrospective study with six ML algorithms.	990 patients across five centers; 71 additional pediatric patients for external validation.	6 machine learning algorithms trains including RF, SVR, GBR, LR, DTR, Extreme GBR, trained on demographics/airway parameters.	Random Forest performed best, with uncuffed ETT MAE 0.275 mm and RMSE 0.349 mm and cuffed ETT MAE 0.243 mm and RMSE 0.310 mm, and it outperformed traditional formulas	Ten-fold cross-validation; external validation using additional 71 patient cohort	Uncuffed MAE 0.275 mm RMSE 0.349 mm and cuffed MAE 0.243 mm RMSE 0.310 mm
Jalali et al, 2016 [15]	Automatic Detection of Endotracheal Intubation During the Anesthesia Procedure	Retrospective algorithm design using wavelet analysis.	600 pediatric surgical cases (1-18 years).	Wavelet analysis of EtCO2 and tidal volume.	Exact intubation time detected in 547 of 600 cases and detected within 1 minute in 96% of cases.	Clinical validation with respiratory data and comparison to recorded intubation time.	Exact detection 547 of 600 which is 91%, detection within 1 minute 96%, four cases failed to converge, and 32 cases detected within three samples of the true time and considered clinically reliable.
Gálvez et al, 2017 [16]	Neural Network Classifier for Automatic Detection of Airway Management Techniques	Retrospective observational study.	900 patients categorized by airway device (300 Mask, 300 LMA, 300 ETT)	Five-layer NN vs. boosted trees/SVM.	NN accuracy: 95.8% vs. SVM (88.3%), boosted trees (91.4%).	5-fold cross-validation.	Accuracy 95.8%, sensitivity 97.5%, specificity 96.3%, and confusion matrix reported for mask versus invasive device classification.
Gray et al, 2023 [17]	A machine learning approach for decision support and risk stratification of pediatric perioperative patients based on the APRICOT dataset	Prospective observational multicenter cohort	30,325 records (0-16 years).	Used gradient boosting model and generated separate models using booking and day-of-surgery feature sets.	Produced two gradient-boosting models to identify low-risk ASA I–III children; AUROC 0.618 (booking) and 0.722 (day-of-surgery) with NPVs >95%.	Internal validation via stratified 70:30 train:test split and test-set evaluation	AUROC: 0.618 (booking), 0.722 (day-of-surgery).
							Booking model (test set): Accuracy 95.2%, Sensitivity 1.2%, Specificity 99.6%, PPV 11.9%, NPV 95.6%.
							Day-of-surgery model (test set): Accuracy 95.4%, Sensitivity 2.7%, Specificity 99.7%, PPV 30.6%, NPV 95.7%.
Yu et al, 2023 [18]	Predicting pediatric emergence delirium using data-driven machine learning applied to electronic health record dataset at a quaternary care pediatric hospital	Single-center retrospective cohort study	54,776 encounters, 43,830 patients (2-13 years).	Nested stratified 10-fold cross validation of LASSO logistic regression, ridge logistic regression, Random Forest, and extreme gradient boosting models using perioperative EHR variables	Emergence delirium occurred in 4,356 encounters for an overall incidence of 8%, with similar discrimination across models with AUROC ranging from 0.74 to 0.75 and PPV ranging from 0.13 to 0.14 at a sensitivity level of 0.8	Internal validation using nested stratified 10-fold cross validation with repeated holdout folds	AUROC 0.74 to 0.75, AUPRC about 0.18 to 0.20 across models and time points, and in testing for the LASSO model through PACU AUROC 0.74 with sensitivity 0.81, specificity 0.54, and PPV 0.13.

The included studies were primarily retrospective cohort or observational studies, with one simulation-based study and a few review articles. Most studies comparing AI/ML models to traditional formulas, such as Shim et al. (2021, 2023) [8,9] and Kim et al. [11], for endotracheal tube (ETT) size and depth, used retrospective datasets, often from the same populations used to derive the conventional formulas. Only Zhou et al. performed external validation across multiple centers, showing slightly lower but still high model accuracy [14]. These designs indicate that while AI/ML models outperform traditional methods within these datasets, prospective, multi-center studies are needed to confirm generalizability and clinical applicability.

AI Applications in Anesthesia

ETT depth and size prediction: Four retrospective cohort studies focused on refining ETT selection using ML models. Kim et al. analyzed data from 37,057 pediatric surgeries using gradient boosted trees trained on age, height, and weight, reducing the mean error to 0.7 cm compared to the 1.2-1.3 cm error range of standard age-based formulas [11]. The model also demonstrated exact-match accuracy ranging from 58.2% to 70.1%. Shim et al. (2021) examined data from 859 pediatric surgical patients under seven years and compared multiple ML algorithms (random forest, elastic net, SVMs, and artificial neural networks) trained on patient age, height, and weight [8]. The random forest model achieved the best performance, accurately predicting appropriate ETT placement in 79.0% of patients, which was statistically significantly higher than both the height-based method (44.4%) and the age-based method (66.9%). The advantage of the random forest model is that it accounts for multiple variables in its prediction, rather than just age or height in isolation. Shim et al. (2023) examined data from 1,436 pediatric intubations, incorporating chest X-ray measurements and elastic net regression to predict ETT depth [9]. Their model was able to predict the appropriate depth of the ET tube 83% of the time, reducing the risk of shallow or deep endotracheal intubations. Zhou et al. performed a retrospective study including 990 pediatric patients and tested six ML models, focusing on ETT size rather than depth [14]. The random forest model performed the best in minimizing prediction error for both uncuffed ETT size (MAE = 0.275 mm and root mean square error (RMSE) = 0.349 mm) and cuffed ETT size (MAE = 0.243 mm and RMSE = 0.310 mm). However, it is important to note that these models were developed and validated using predominantly homogeneous populations, which may limit their generalizability across diverse ethnic and anatomical groups, particularly given known differences in airway anatomy across populations.

Intraoperative monitoring and hypoxemia prediction: Park et al. developed machine learning models to predict intraoperative hypoxemia in pediatric patients using intraoperative monitor and ventilator data recorded every two seconds, with the outcome defined as oxygen saturation below 95% and the prediction task framed as forecasting whether hypoxemia would occur in the upcoming one minute based on the prior one-minute segment of data. Input variables included patient demographics (age, sex, height, and weight) and real-time physiologic parameters (oxygen saturation (SpO2), end-tidal carbon dioxide (EtCO2), fraction of inspired oxygen (FiO2), tidal volume (TV), and peak inspiratory pressure (PIP)) extracted every two seconds [12].

They screened 13,130 pediatric anesthesia records for model development, including 1,540 patients with hypoxemia and 2,367 hypoxemia episodes. Gradient boosting machine, long short-term memory, and transformer models were trained and compared. In the development test dataset, gradient boosting achieved an AUROC of 0.904, compared with 0.843 for long short-term memory and 0.897 for the transformer model. In temporal holdout validation using later cases from November 2020 to January 2021, gradient boosting achieved an AUROC of 0.939 with a sensitivity of 0.855 and a specificity of 0.807, exceeding long short-term memory AUROC 0.904 and transformer AUROC 0.929. The holdout cohort included 1,510 records with 200 hypoxemic patients and 289 hypoxemia episodes, representing a relatively low event rate that should be considered when interpreting model performance metrics [12].

Bi-level positive airway pressure (BIPAP) failure prediction: Im et al. conducted a retrospective cohort study of 630 pediatric BiPAP sessions, with 175 failures for a 27.7% failure rate, defining BiPAP failure as escalation to invasive mechanical ventilation within 48 hours of BiPAP termination [10]. They trained a long short-term memory recurrent neural network using 301 electronic medical record variables including vital signs (heart rate, respiratory rate, oxygen saturation), laboratory results, medications (antibiotics, vasopressors, inotropes, diuretics, sedatives), respiratory support parameters (supplemental oxygen and BiPAP settings), invasive procedures, radiography findings, and nursing assessments to generate time updated predictions and compared performance with logistic regression-based models and a reference oxygenation ratio model.

The Long Short-Term Memory Recurrent Neural Network (LSTM-RNN) model was a deep learning architecture designed to process time-series data sequentially, incorporating information from previous time points with newly available inputs to continuously update predictions as a patient's clinical status evolved on BiPAP. This model was compared against two logistic regression models: one using variables similar to the HACOR (Heart rate, Acidosis, Consciousness, Oxygenation, and Respiratory rate) scale and another using the same 301 electronic medical record (EMR) variables as the LSTM-RNN but without the temporal modeling capabilities. Model evaluation used an 80% training and 20% holdout test split, with all sessions from a given patient kept within the same split. In the overall cohort, the LSTM-RNN model's discrimination was AUROC 0.81 at six hours and 0.84 at 24 hours, and in the hypoxemic subgroup, it was AUROC 0.88 at six hours and 0.86 at 24 hours. When operating at a number needed to alert of 2, the model identified nearly 80% of BiPAP failures within six hours. The Number Needed to Alert of 2 indicates that for every two alerts generated, one would correspond to a true BiPAP failure, providing clinically relevant context about the alert burden clinicians would experience using this model. Among failure sessions, the median time on BiPAP prior to intubation was 32.8 hours [10].

Pain assessment and postoperative management: Gude et al. performed a secondary analysis of prospectively collected inpatient pain assessments in children aged two to four years after tonsil surgery to derive a shorter rule for identifying indications for rescue analgesia [13]. They analyzed 1067 pain intensity assessments from 149 children through postoperative day three, defining rescue medication indication as Children's and Infants Postoperative Pain Scale (CHIPPS) score of at least 4 or Parents Postoperative Pain Measure - German (PPPM-D) score of at least 6. CHIPPS is a five-item observational behavioral pain scale that assesses crying, facial expression, posture of trunk, posture of legs, and motor restlessness, with each item scored 0-2 points for a total score range of 0-10, where a score of 4 or higher indicates analgesic demand. The PPPM-D is a 15-item questionnaire completed by parents that assesses behavioral changes in children (such as whining, crying, playing less, acting worried, having less energy, refusing to eat, groaning, and other behaviors), with each item answered as yes or no; a total score of 6 or more (out of 15) indicates clinically significant pain requiring intervention.

Rescue medication indications occurred in 213 assessments, which is 19.96% of all assessments, with 54 meeting the CHIPPS threshold only, 143 meeting the PPPM-D threshold only, and 16 meeting both. Using Boruta feature selection, PPPM-D score and CHIPPS score were equally informative, and multiple PPPM-D items and time of assessment were also identified as associated with rescue medication indication. Symbolic regression then produced a four-predictor decision rule with a cross-validated accuracy of 94.94%, termed the Bochum Postoperative Pain Instrument (BPPI), indicating rescue medication when CHIPPS is at least 4, or when the child has less energy than usual, and either cries more easily than usual or groans or moans more than usual [13].

Yu et al. developed machine learning models to predict pediatric emergence delirium using single-center EHR data from February 2015 through December 2019 at the Children’s Hospital of Philadelphia, including patients aged two years to under 13 years undergoing procedures at the main hospital or ambulatory surgery center [18]. Emergence delirium was defined as a Watcha score of 3 or 4 recorded at any time during PACU recovery. The dataset included 54,776 encounters across 43,830 patients, with emergence delirium occurring in 4,356 encounters for an overall incidence of 8%. A total of 75 variables were extracted from the EHR, including demographic variables (age, weight, height, sex, race, ethnicity), American Society of Anesthesiologists (ASA) physical status (PS), gestational age at birth, history of prior surgery, surgical procedure type, International Classification of Diseases (ICD) codes for comorbidities (developmental delay, seizure disorder, asthma/reactive airway disease, obstructive sleep apnea, attention deficit/hyperactivity disorder, and autism spectrum disorder), postoperative disposition, surgical facility type, fasting times, preoperative vital signs (heart rate and blood pressure), sedative premedications, patient behavior at induction, parental presence at induction, anesthesia induction type, intraoperative medications, airway management, regional anesthesia techniques, nursing assessments, and event timestamps.

Four models were trained and tested using nested stratified 10-fold cross-validation, including least absolute shrinkage and selection operator (LASSO) logistic regression, ridge logistic regression, random forest, and extreme gradient boosting. Model discrimination was similar across approaches, with AUROC ranging from 0.74 to 0.75, and at a sensitivity level of 0.8, specificity ranged from 0.50 to 0.57, with positive predictive value (PPV) ranging from 0.13 to 0.14. In the LASSO model performance reported through the post-anesthesia care unit (PACU), testing metrics included AUROC 0.74 with sensitivity 0.81, specificity 0.54, and PPV 0.13 [18].

Airway device classification and intubation detection: Jalali et al. developed a wavelet-based algorithm to automatically detect the timing of endotracheal intubation during anesthesia using de-identified ventilator-derived respiratory monitoring data from 600 pediatric surgical patients aged 1-18 years, with end-tidal carbon dioxide, tidal volume, and peak inspiratory pressure sampled every 15 seconds [15]. The method applied wavelet analysis to identify characteristic signal changes around intubation, using end-tidal carbon dioxide to flag candidate time periods and then using tidal volume or peak inspiratory pressure to determine the exact intubation time. In evaluation against recorded intubation times, the algorithm detected the intubation time exactly in 547 of 600 cases and within one minute in 96% of cases, with four cases failing to converge and 32 cases detected within three samples of the true time and considered clinically reliable [15].

Gálvez et al developed a five-layer neural network classifier to automatically detect airway management techniques using de-identified anesthesia records from 900 pediatric patients, with 300 cases each managed with mask ventilation, laryngeal mask airway, or endotracheal tube [16]. The primary classification task was noninvasive mask ventilation versus invasive airway management, defined as laryngeal mask airway or endotracheal tube, using statistical features derived from respiratory monitoring parameters, including EtCO2, TV, PIP, and respiratory rate. Model performance was evaluated with five-fold cross-validation, and the neural network achieved 95.8% accuracy with a sensitivity of 97.5% and a specificity of 96.3%, outperforming boosted trees with 91.4% accuracy and support vector machines with 88.3% accuracy.

Perioperative risk prediction and monitoring: Gray et al. developed machine learning models to classify pediatric patients as low risk for severe perioperative adverse events at two clinically meaningful timepoints: Surgical booking and after day-of-surgery anesthetic assessment [17]. Their APRICOT (Anaesthesia PRactice In Children Observational Trial) prospective multicenter dataset was used to train and test models, which consisted of a large European study conducted by 261 institutions in 33 European countries from April 2014 to January 2015. Data collected included patient demographics such as age, sex, and weight; preoperative assessment variables including history of wheezing, prematurity, family smokers, previous anesthetic complications, medication use, metabolic or genetic disorders or neurological impairment, asthma, atopy, allergy, snoring, ASA-PS score, recent flu or cold, fever, and surgical urgency; intraoperative management details such as anesthesia consultation, type and experience of anesthesia provider, sedative premedication, parental presence at induction, monitoring type, induction type, airway interface type, fluids administration, regional anesthesia use, and ventilation type; and postoperative recovery information.

The primary endpoint was defined as perioperative critical events encompassing respiratory, cardiac, allergic, or neurological complications requiring immediate intervention that led or could have led to major disability or death, with drug errors excluded as they do not relate to the underlying patient condition. Using APRICOT data limited to first procedures in ASA PS I to III patients and excluding drug error events, they analyzed 30,325 records with an adverse event rate of 4.43%. They used a stratified 70:30 split, meaning 70% of cases were used to train the model and 30% were held out to test it, while keeping the same proportion of adverse events and non-events in both groups, and gradient boosting was the best-performing approach. Model performance emphasized negative predictive value (NPV), with NPVs greater than 95% and an AUROC of 0.618 for the booking model and 0.722 for the day-of-surgery model. On the test set, the booking model performance showed an accuracy of 95.2%, a sensitivity of 1.2%, a specificity of 99.6%, a PPV of 11.9%, and an NPV of 95.6%. The day-of-surgery model showed accuracy of 95.4%, sensitivity of 2.7%, specificity of 99.7%, PPV of 30.6%, and NPV of 95.7% [17].

Risk of Bias

Risk of bias was assessed using the Prediction Model Risk of Bias Assessment Tool (PROBAST), which evaluates four domains: participants, predictors, outcome, and analysis [19]. While PROBAST is designed for clinical prediction models, it was applied pragmatically to assess bias domains across heterogeneous AI studies, recognizing its limitations for classification and signal-detection tasks such as intubation detection and airway device classification. Overall risk of bias across included studies was low to moderate. Most studies used retrospective single-center datasets with internal validation only, resulting in moderate risk (some concerns) in the participants and analysis domains due to limited generalizability and potential overfitting. Predictor and outcome definitions were consistently clear and objectively measured, resulting in low risk in these domains across studies. Two studies [14,17] incorporated multicenter data and external or prospective validation and were therefore judged to have low overall risk of bias. The complete risk of bias assessment for all included studies is presented in Table 4.

**Table 4:** Risk of Bias Assessment Using PROBAST Framework PROBAST = Prediction Model Risk of Bias Assessment Tool

Study (Author, Year)	Selection Bias	Performance Bias	Detection Bias	Attrition Bias	Reporting Bias	Overall Risk of Bias	Justification
Shim et al., 2021 [8]	Some concerns	Low	Low	Low	Low	Some concerns	Retrospective single-center cohort; no external validation, but objective outcome measures and appropriate internal cross-validation.
Shim et al., 2023 [9]	Some concerns	Low	Low	Low	Low	Some concerns	Retrospective design with internal validation only; outcome definition objective; potential population-specific model fitting.
Im et al., 2022 [10]	Some concerns	Low	Low	Low	Low	Some concerns	Retrospective cohort; model trained and tested on separate splits but no external validation; objective outcome (BiPAP failure).
Kim et al., 2023 [11]	Some concerns	Low	Low	Low	Low	Some concerns	Large retrospective dataset with temporal internal validation; no external validation; objective airway outcome measures.
Park et al., 2023 [12]	Low	Low	Low	Low	Low	Low	Large dataset with temporal holdout validation; objective physiologic outcome; robust internal validation strategy.
Gude et al., 2024 [13]	Some concerns	Low	Some concerns	Low	Low	Some concerns	Secondary analysis of prospectively collected data; pain outcomes partly subjective; internal cross-validation only.
Zhou et al., 2022 [14]	Low	Low	Low	Low	Low	Low	Multi-center dataset with external validation cohort; objective measurement of ETT size prediction.
Jalali et al., 2016 [15]	Some concerns	Low	Low	Low	Low	Some concerns	Retrospective algorithm development; single-center dataset; objective signal-based outcome; limited external validation.
Gálvez et al., 2017 [16]	Some concerns	Low	Low	Low	Low	Some concerns	Retrospective single-center data; internal cross-validation only; objective classification outcome.
Gray et al., 2023 [17]	Low	Low	Low	Low	Low	Low	Prospective multi-center cohort (APRICOT); standardized data collection; prespecified outcomes and validation.
Yu et al., 2023 [18]	Some concerns	Low	Low	Low	Low	Some concerns	Large single-center retrospective EHR dataset; nested cross-validation; no external validation.

Discussion

Summary of Key Findings

This structured narrative review demonstrates that AI applications show promise for enhancing the precision, efficiency, and safety of pediatric anesthesia care through improved decision support systems based on predictive performance in predominantly retrospective studies. The findings highlight the potential role AI can play in pediatric anesthesia, showing improved predictive performance in several areas. AI-driven models have demonstrated improved statistical performance compared with traditional approaches in several areas, including optimizing endotracheal tube sizing, predicting intraoperative hypoxemia, and refining pain management strategies. The ability of machine learning algorithms to analyze vast amounts of perioperative data enables early identification of risks, contributing to improved patient safety and more precise anesthetic management. These models span a diverse range of algorithms, including gradient boosted trees, random forests, long short-term memory networks (LSTM-RNNs), SVMs, symbolic regression, and wavelet-based signal analysis, each applied to different challenges.

One significant benefit of AI in pediatric anesthesia is its capacity to enhance airway management. Studies have shown that AI models, particularly gradient boosted trees and random forest, significantly reduce misplacement errors in ETT placement by incorporating demographic and physiological variables that traditional formulas may overlook [1,20,21]. Accurate ETT placement is crucial in pediatric patients due to their smaller and more delicate airways, making AI-assisted decision-making a valuable tool in anesthesia planning. The ability of AI to predict optimal insertion depth based on individual patient characteristics, rather than relying solely on weight or age-based estimates, presents a notable advancement in pediatric anesthetic care. In addition to placement depth, AI models have also improved the precision of ETT size selection. For instance, Zhou et al. demonstrated that random forest models could minimize error in both cuffed and uncuffed tube size predictions with mean absolute errors below 0.3 mm [14]. These findings underscore the importance of integrating AI into airway management, especially in pediatric patients, where small miscalculations can lead to significant complications.

Another critical application of AI within the anesthesia setting is in intraoperative monitoring, particularly in the early detection of hypoxemia. Traditional monitoring methods rely on threshold-based alarms, which may not provide sufficient warning before a critical event occurs [12]. Additional studies support that pulse oximetry measurements can lag several minutes behind the onset of hypoventilation or apnea, potentially delaying critical interventions [22]. AI-based predictive models, such as LSTM networks, have demonstrated the capability to forecast hypoxemia episodes before clinical manifestation, allowing anesthesiologists to intervene proactively and limit end-organ compromise [12,14]. This predictive capacity is particularly relevant in pediatric patients, where rapid physiological changes can lead to sudden desaturation events. AI-driven monitoring systems can analyze real-time oxygen saturation trends, ventilation parameters, and hemodynamic signals, offering anesthesiologists enhanced situational awareness and decision-making support.

Beyond intraoperative management, AI has also been applied to postoperative pain assessment, addressing a long-standing challenge in pediatric anesthesia. Traditional pain assessments rely heavily on subjective measures, which can be inconsistent and vary based on the clinician's experience. AI-powered pain scoring models, utilizing Boruta feature selection and symbolic regression, have been shown to achieve high accuracy in evaluating pain levels in pediatric patients [3]. Gude et al. reported that their BPPI achieved 94.94% accuracy in predicting the need for rescue analgesics in toddlers after tonsil surgery, highlighting its potential for improving ease of use and clinical efficiency [13].

These models provide a more standardized and objective assessment, reducing variability and ensuring more appropriate analgesic administration. By incorporating behavioral cues, facial expressions, and physiological markers, AI models can enhance pain management strategies and contribute to improved recovery outcomes. Other emerging AI applications in pediatric anesthesia include automated airway device classification and real-time intubation detection, as shown by Jalali et al. [15] and Gálvez et al. [16] in their studies. These varied applications reflect the breadth of AI integration potential across all stages of anesthesia care, from induction to recovery.

Several studies included in this review compared AI/ML models to traditional formulas for predicting endotracheal tube (ETT) size and depth. Shim et al. [8,9] and Kim et al. [11] reported higher rates of correct placement with machine learning algorithms compared to age- or height-based formulas. However, most comparisons used retrospective datasets derived from the same populations that informed the traditional formulas, so reported high performance may reflect dataset-specific optimization rather than true superiority, limiting generalizability to other pediatric populations. Only a few studies, such as Zhou et al., performed external validation with independent, multi-center datasets, which showed slightly reduced accuracy compared to internal validation [14]. These findings underscore the need for prospective, multi-center studies to rigorously evaluate AI/ML models against established formulas, ensuring reliable clinical implementation across diverse pediatric populations.

Comparative Effectiveness of AI vs. Traditional Methods

The studies included in this review indicate that AI-based approaches demonstrate improved predictive performance within studied datasets, with models in several studies showing signals of greater statistical accuracy and predictive capabilities compared with traditional methods. However, it is important to distinguish between statistical performance in retrospective datasets and clinical effectiveness in real-world practice settings.

AI models have demonstrated meaningful improvements in ETT prediction accuracy. For insertion depth, Kim et al. found that gradient boosted trees reduced mean absolute error from 1.2-1.3 cm with traditional formulas to 0.71-0.72 cm, representing approximately a 40-45% reduction in prediction error [11]. For ETT size selection, Zhou et al. demonstrated that random forest models achieved mean absolute errors below 0.3 mm for both cuffed and uncuffed tubes, outperforming traditional formula-based approaches [14]. Traditional methods rely on generalized population-based formulas that fail to account for individual patient variability, whereas AI incorporates multiple patient-specific parameters to enhance precision [2].

In intraoperative monitoring, AI-driven hypoxemia prediction models demonstrated AUROC values ranging from 0.90 to 0.94, indicating substantially better discrimination than would be expected from simple single-parameter thresholds. Though Park et al.'s study did not perform any direct comparison to conventional alarm systems, they demonstrated that a GBM model could predict hypoxemia 60 seconds in advance, allowing clinicians a proactive window for intervention. Additionally, LSTM models showed enhanced performance in hypoxemic subgroups, highlighting AI’s ability to manage dynamic physiologic changes in real time [12]. Traditional systems often react only after oxygen desaturation begins, limiting the opportunity for early clinical action. In contrast, AI systems evaluate multiple real-time inputs, including SpO₂ trends, ventilation metrics, and hemodynamics, to provide anticipatory alerts [2].

For postoperative pain assessment in toddlers after tonsil surgery, Gude et al. applied Boruta feature selection and symbolic regression to derive the BPPI, a four-variable rule combining CHIPPS and selected PPPM-D items [13]. In a secondary analysis of 1,067 pain assessments, this BPPI logic achieved a cross-validated accuracy of 94.94% for identifying indications for rescue analgesia, comparable to the full 15-item PPPM-D threshold while using fewer items. Conventional behavioral scales such as CHIPPS and related tools like FLACC can show low concordance and risk misjudging pain, particularly in less expressive children, whereas BPPI focuses on the PPPM-D items most strongly associated with the need for additional analgesia and may help streamline and standardize postoperative pain assessment [13]. Traditional pain assessment methods often rely on observational scoring or patient self-report, which can introduce subjectivity and variability in opioid administration and recovery outcomes [3]. Such variability may also stem from differences in provider specialties and attitudes toward opioid prescribing [23].

For perioperative risk, Gray et al showed that an AI model can look strong on some performance measures but still miss many true events. In APRICOT, perioperative adverse events were uncommon at 4.43%, and both models had high overall accuracy and high negative predictive value, meaning most patients predicted as not having an event truly did not have an event [17]. However, sensitivity was low at 1.2% for the booking model and 2.7% for the day-of-surgery model, which means the models identified only a small fraction of the patients who actually experienced an adverse event. When comparing AI models to traditional approaches, these results highlight why it is important to report multiple metrics together, including sensitivity, specificity, positive predictive value, and negative predictive value, rather than relying only on AUROC or accuracy.

Interpretation of Performance Metrics and Clinical Utility

While the studies reviewed report a range of performance metrics, including AUROC, accuracy, sensitivity, specificity, and positive and negative predictive values, interpretation of these metrics requires careful consideration of clinical context, particularly for low-incidence pediatric outcomes. AUROC values, while useful for assessing discriminative ability, can be misleading when evaluated in isolation for imbalanced datasets. For instance, the hypoxemia prediction study by Park et al. reported impressive AUROC values of 0.904 to 0.939, yet the relatively low incidence of hypoxemia events (1,540 patients with hypoxemia out of 13,130 total cases, approximately 11.7%) means that AUROC alone does not fully capture the clinical utility of the model in practice [12]. Similarly, in the perioperative risk prediction study by Gray et al., we appropriately noted that despite high AUROC values (0.618-0.722) and accuracy (>95%), the models demonstrated very low sensitivity (1.2-2.7%), identifying only a small fraction of patients who actually experienced adverse events [17]. This highlights the critical importance of examining multiple performance metrics in concert, particularly sensitivity, specificity, PPV, and NPV, rather than relying on AUROC or overall accuracy alone. The Number Needed to Alert (NNA) metric reported by Im et al. for BiPAP failure prediction (NNA of 2 to identify nearly 80% of failures) provides useful clinical context by quantifying the trade-off between alert burden and detection rate, though such clinically-oriented metrics remain underreported across the literature [10]. Future AI research in pediatric anesthesia should prioritize reporting comprehensive performance metrics appropriate for the clinical question, including calibration statistics and, ideally, decision-curve analysis or other measures of clinical utility that account for the costs and benefits of different decision thresholds. Without such context, even statistically impressive models may have limited practical value in clinical decision-making.

Challenges and Limitations

Despite these promising developments, several challenges hinder the widespread clinical adoption of AI in pediatric anesthesia. One primary limitation is the reliance on retrospective data for training and validation. Many AI models have been developed and tested on historical datasets from single institutions, raising concerns about their generalizability across varying populations and clinical settings. Additionally, it should be noted that the current literature on AI applications specific to pediatric anesthesia remains limited, as reflected by the 11 studies meeting our inclusion criteria, highlighting the nascent state of this field. While adult anesthesia and perioperative AI literature is more extensive and may inform future pediatric research directions, extrapolations from adult populations should be interpreted as contextual background rather than directly applicable to pediatric practice, given the unique physiological, anatomical, and developmental considerations inherent to pediatric populations. This is because there are anatomical differences in airways across a variety of populations that single data sets will not adequately account for. For example, Asian populations tend to have smaller upper airway bony dimensions and more craniofacial skeletal restriction in comparison to white populations. White populations also often have larger upper airway soft tissue volumes [24-26]. Training ML models on non-diverse datasets not only limits generalizability but also reinforces existing biases.

The generalizability of AI models in pediatric anesthesia is significantly constrained by the interconnected issues of retrospective design, population homogeneity, and algorithmic bias. Most studies in this review were conducted at single centers with relatively homogeneous patient populations, raising concerns about model transportability to diverse clinical settings. The risk of algorithmic bias is particularly concerning in airway management applications, where anatomical differences across ethnic populations are well established. For instance, the ETT sizing and placement models discussed earlier were predominantly developed in Korean or other specific populations, and their performance may deteriorate when applied to patients with different craniofacial characteristics. This population-specific optimization, combined with the retrospective nature of most studies, creates a substantial risk that models will not perform as well in external validation or real-world implementation, especially in low-resource settings where patient populations may differ significantly from the training data. Addressing these limitations will require prospective multicenter studies with intentionally diverse patient cohorts and rigorous external validation across different demographic and geographic settings.

The potential for inadvertent bias further limits the generalizability of the findings. The datasets that AI models are based on can carry inherent biases that AI models will learn from, absorb, and reinforce without appropriate protective measures. Harvard Medical School outlined three in 2024, including data diversification, continuous monitoring of data, and utilizing interdisciplinary collaborations [27]. To ensure reliability and effectiveness, AI models must be externally validated using multi-center datasets that include diverse patient demographics. Additionally, prospective trials should be conducted to assess the real-world performance of AI-driven decision support tools in clinical practice [28].

Another major challenge is the integration of AI into existing anesthesia workflows. While AI models offer substantial improvements in predictive accuracy, their implementation requires seamless interoperability with electronic health record systems and anesthesia monitoring platforms. Many hospitals and surgical centers operate with heterogeneous software and hardware systems, making the integration process complex and resource-intensive. The development of standardized frameworks and AI-compatible clinical decision support systems will be essential in facilitating the transition from research to routine practice.

Clinician understanding also remains paramount in the integration of AI into clinical practice. ML models should emphasize transparency so that clinicians remain able to understand the decision-making process done by ML models, maintaining their ability to troubleshoot and notice any inaccuracies. Black-box models should be avoided in this setting, as these models are not transparent to the person utilizing them and are therefore making autonomous and unmonitored decisions [29].

ML models should also prioritize equitable access so as not to widen existing health disparities that impact lower socioeconomic demographics. The cost of incorporating ML models into healthcare should be considered, as if the tools are too costly, only resource-rich areas will be able to afford to integrate them into practice. For this reason, it is paramount to prioritize cost-effective models or develop other ways to ensure equitable access. Lawmakers have begun to address the possible inequities that ML models may introduce, but continued efforts on both the state and federal levels will be required to prevent them from materializing [30].

Regulatory considerations also play a crucial role in AI adoption. As AI models become more prevalent in clinical decision-making, ensuring patient safety and maintaining clinician oversight remains paramount. Regulatory agencies such as the United States Food and Drug Administration (FDA) and Conformité Européenne (CE) must establish clear guidelines for the approval and monitoring of AI-based medical technologies. Ethical concerns, including data privacy, informed consent, and the potential for bias in AI models, must also be addressed. AI algorithms are only as reliable as the data on which they are trained, and biases in training datasets can lead to disparities in model performance across different patient populations.

Future Research

Future research should focus on addressing these limitations by conducting large-scale prospective studies, improving model explainability, and fostering collaboration between AI developers and clinical practitioners. Expanding the use of multi-center trials is necessary to validate AI models across diverse patient populations and clinical settings. Efforts should also be made to integrate AI into real-time clinical decision support systems that work seamlessly within existing hospital infrastructures. Additionally, refining AI-based predictive models to minimize bias and increase interpretability for anesthesiologists will be crucial. AI research should also prioritize the development of hybrid models that combine AI-driven insights with clinician expertise to enhance decision-making while ensuring patient safety. Further investigations into regulatory frameworks and ethical considerations will be necessary to facilitate widespread AI adoption in pediatric anesthesia. Efforts should also be made to develop hybrid AI-human decision-making models, where AI serves as a supportive tool rather than a replacement for anesthesiologists. Such models can enhance trust in AI recommendations and ensure that clinical expertise remains central to patient care. Additionally, further advancements in deep learning techniques and reinforcement learning may enhance AI’s capability to adapt to dynamic perioperative environments, making it even more of a valuable asset in pediatric anesthesia.

## Conclusions

AI and ML have demonstrated substantial potential to transform pediatric anesthesia practice by showing improved predictive performance that may enhance clinical decision-making, patient safety, and perioperative outcomes, though clinical validation remains needed. This narrative review synthesized evidence from 11 studies examining diverse applications across the continuum of anesthetic care, from preoperative planning to postoperative management. The findings consistently demonstrate that AI-driven models show improved predictive performance compared with traditional clinical methods in retrospective analyses of optimal endotracheal tube sizing and placement, predicting intraoperative complications such as hypoxemia, assessing postoperative pain, and stratifying perioperative risk. These observed improvements in predictive performance stem from AI's capacity to integrate multiple patient-specific variables simultaneously and identify complex patterns that may not be apparent with conventional population-based formulas and threshold-based monitoring systems. However, these findings are based primarily on retrospective single-center studies, and prospective validation in diverse clinical settings is needed to establish clinical effectiveness and real-world impact on patient outcomes. 

Most current evidence derives from retrospective single-center studies with limited external validation, raising concerns about model generalizability across diverse patient populations and clinical settings. The risk of algorithmic bias, particularly when models are trained on homogeneous datasets, threatens to perpetuate or exacerbate existing healthcare disparities if not proactively mitigated through inclusive data collection and continuous monitoring. Additionally, successful integration of AI into clinical workflows requires not only technical interoperability with existing electronic health record and monitoring systems but also transparent model architectures that maintain clinician understanding and oversight of decision-making processes. Regulatory frameworks must evolve to establish clear standards for AI approval and ongoing surveillance while addressing ethical considerations, including data privacy, informed consent, and equitable access to these technologies. Future research priorities should emphasize prospective multicenter validation trials, development of interpretable hybrid AI-human decision support models, and implementation science studies that examine real-world clinical adoption barriers and facilitators. By addressing these challenges through collaborative efforts among AI developers, clinical practitioners, regulatory bodies, and healthcare institutions, the pediatric anesthesia community can effectively integrate artificial intelligence to deliver more precise, efficient, and equitable care to vulnerable pediatric populations.
